# *Aedes aegypti* larval development and pupal production in the FAO/IAEA mass-rearing rack and factors influencing sex sorting efficiency

**DOI:** 10.1051/parasite/2020041

**Published:** 2020-06-18

**Authors:** Wadaka Mamai, Hamidou Maiga, Nanwintoum Séverin Bimbilé Somda, Thomas Wallner, Anna Konczal, Hanano Yamada, Jérémy Bouyer

**Affiliations:** 1 Insect Pest Control Laboratory, Joint FAO/IAEA Division of Nuclear Techniques in Food and Agriculture 1400 Vienna Austria; 2 Institut de Recherche Agricole pour le Développement (IRAD) PO Box 2123 Yaoundé Cameroun; 3 Institut de Recherche en Sciences de la Santé/Direction Régionale de l’Ouest (IRSS/DRO) 01 PO Box 545 Bobo-Dioulasso Burkina Faso; 4 Laboratoire d’Entomologie Fondamentale et Appliquée (LEFA), Université Joseph Ki-Zerbo 03 PO Box 7021 Ouagadougou Burkina Faso; 5 CIRAD, UMR ASTRE CIRAD-INRA “Animals, Health, Territories, Risks and Ecosystems” Campus International de Baillarguet 34398 Montpellier Cedex 05 France

**Keywords:** Vectors, Mosquitoes, Sterile insect technique, Female contamination, Pupal production, Fay–Morlan glass plate separator

## Abstract

The production of a large number of mosquitoes of high biological qualities and reliable sex sorting before release are key challenges when applying the sterile insect technique as part of an area-wide integrated pest management approach. There is a need to fully evaluate the production capacity of the equipment developed in order to plan and maintain a daily production level for large-scale operational release activities. This study aimed to evaluate the potential use of the FAO/IAEA larval rearing unit for *Aedes aegypti* and the subsequent female contamination rate after sex sorting with a Fay–Morlan glass separator. Trays from each rack were tilted and their contents sorted either for each individual tray or after mixing the content of all trays from the rack. The pupal production and the female contamination rate were estimated with respect to day of collection, position of the tray, type of pupae collection, and sorting operator. Results showed significant daily variability of pupal production and female contamination rate, with a high male pupal production level achieved on the second day of collection and estimated female contamination of male pupae reached around 1%. Neither tray position nor type of pupae collection affected the pupal production and female contamination rate. However, the operator had a significant effect on the female contamination rate. These results highlight the need to optimize pupal production at early days of collection and to develop a more effective and automated method of sex separation.

## Abstract (fr)

La production d’un grand nombre de moustiques de haute qualité biologique et le tri des sexes avant les lâchers sont des défis clés lors de l’application de la technique des insectes stériles, dans le cadre d’une approche de lutte intégrée contre les ravageurs à l’échelle d’une zone. Il est nécessaire d’évaluer pleinement la capacité de production des équipements développés afin de planifier et de maintenir un niveau de production quotidien pour les activités de libération opérationnelle à grande échelle. Cette étude visait à évaluer l’utilisation potentielle de l’unité d’élevage larvaire FAO/AIEA pour *Aedes aegypti* et le taux de contamination par des femelles après le tri sexuel avec un séparateur en verre Fay–Morlan. Les plateaux de chaque rack ont été inclinés et leur contenu trié soit pour chaque plateau, soit après avoir mélangé le contenu de tous les plateaux du rack. La production de pupes et le taux de contamination par des femelles ont été estimés en fonction du jour de collecte, de la position du bac, du type de collecte des pupes et de l’opérateur du tri. Les résultats ont montré une variabilité quotidienne significative de la production de pupes et du taux de contamination par des femelles, avec un niveau élevé de production de pupes mâles atteint le deuxième jour de collecte et la contamination estimée des pupes mâles par des femelles a atteint environ 1 %. Ni la position du plateau ni le type de collecte des pupes n’ont affecté la production de pupes et le taux de contamination par des femelles. Cependant, l’opérateur avait un effet significatif sur le taux de contamination par les femelles. Ces résultats mettent en évidence la nécessité d’optimiser la production des pupes dès les premiers jours de la collecte et de développer une méthode de séparation des sexes plus efficace et automatisée.

## Introduction

The sterile insect technique (SIT) is not a new approach as its integrated use in area-wide pest management (AW-PM) has been successful to suppress or even eradicate several major insect pests, such as the New World screwworm *Cochliomyia hominivorax* [42], the tsetse fly *Glossina austeni* [43] and the Mediterranean fruit fly *Ceratitis capitata* [15]. First attempts to use the SIT against mosquitoes date back six decades [35]. In the 1970s, there was much research conducted on the SIT against *Anopheles albimanus* in El Salvador [10, 26, 44] with a significant degree of population suppression. Since then, it went into “eclipse” but has gained increased attention over the past few decades against vectors such as *Anopheles arabiensis*, *Aedes albopictus*, and *Ae. aegypti* [5, 7, 13, 22, 23, 25, 52]. The burden of mosquito transmitted diseases remains enormous despite scaled-up intervention efforts using conventional methods that include habitat management and the use of pesticides which have shown only short-term efficacy due to rapid evolution of insecticide resistance [45]. The recent epidemics of Zika disease in South America in 2015 and 2016 have raised awareness of new complementary and sustainable approaches and SIT appears to be regaining momentum. *Aedes aegypti*, the primary vector of Zika virus worldwide [20, 46] is considered responsible for these recent outbreaks in the Americas. The requirement of producing a large number of mosquitoes of high biological qualities is a key challenge for SIT application. Furthermore, it is required to release only sterile male mosquitoes as bites from females might reduce the acceptability of the technique and increase, to some extent, the risk of transmitting diseases. Hence, the lack of a perfect/efficient sex sorting method is one of the major bottlenecks that delays the large-scale application of this technique [27]. Significant technological and methodological advances have been made at the Insect Pest Control Laboratory (IPCL) of the Joint FAO/IAEA Division of Nuclear Techniques in Food and Agriculture in all of the SIT components, including the development of adult mosquito cages [3, 28, 30, 31], egg quantification method or techniques [29, 50], suitable and cheaper diets for larval feeding [8, 32], handling and quality control devices [11, 12], mass-rearing and irradiation procedures, and guidelines [16, 47]. For the immature stages, trays and racks were developed for rearing of large numbers of larvae [2, 4, 49]. The separation of males and females in many SIT pilot studies is currently performed using methods based on sexual size dimorphism at the pupal stage such as the sieving plates and the Fay–Morlan glass plate separator [5, 22, 37]. Pilot field trials leading to SIT application against *Ae. aegypti* are currently being undertaken in some countries such as Brazil, Cuba, Mauritius, Mexico, Thailand, and Greece [9]. However, implementation on a large-scale of the SIT requires full evaluation of the production capacity of dedicated equipment or technology in order to plan and maintain a daily production level for large-scale operational release activities. The current technology for mosquito larval mass-rearing is based on a system consisting of a rack that can hold up to 50 rearing trays [2, 4, 33, 49]. However, one of the key challenges in mosquito larval mass-rearing is to find the right balance between labor (handling), investment (feed and diet cost), productivity, and quality. The production of this tray-rack system has been evaluated in some facilities for *Ae. albopictus* [4, 49]. The quality and quantity of larval diet, larval density, and environmental conditions have the greatest impact on growth, development, survival, size, and productivity. Therefore, depending on these factors, pupation can take several days. Moreover, depending on the species, the structure of the rack, the height of the trays when stacked within the rack, along with different light intensity received, air movement and lack of an automated larval feeding system, could lead to some variability in larval development and therefore could induce differences in pupae size. In an SIT program or other related male release programs, number, quality and purity of males to be released are of high importance. The question therefore remains whether it is necessary to sort pupae more than once in order to maximize male pupae recovery. What could be the consequence in term of sex sorting efficiency? Evaluating the impact of such factors could contribute to determine the best pupae collection time and sorting method to optimize male pupal production and reduce the female contamination rate in the released male mosquitoes. The objective of this study was to evaluate the potential use of the FAO/IAEA larval rearing unit for *Ae. aegypti* and the subsequent female contamination rate after sex sorting with the Fay–Morlan glass separator. The experimental design was set up to specifically assess (i) the impact of tray position within the rack associated with larval water temperature on pupal production and female contamination rate, (ii) female contamination rates in male pupae when sorting pupae retrieved from individual trays against mixing the contents of all the trays before sorting, and (iii) the effect of sorting operator and day of pupation on female contamination rate.

## Materials and methods

### Mosquito colony

**Figure 1:**
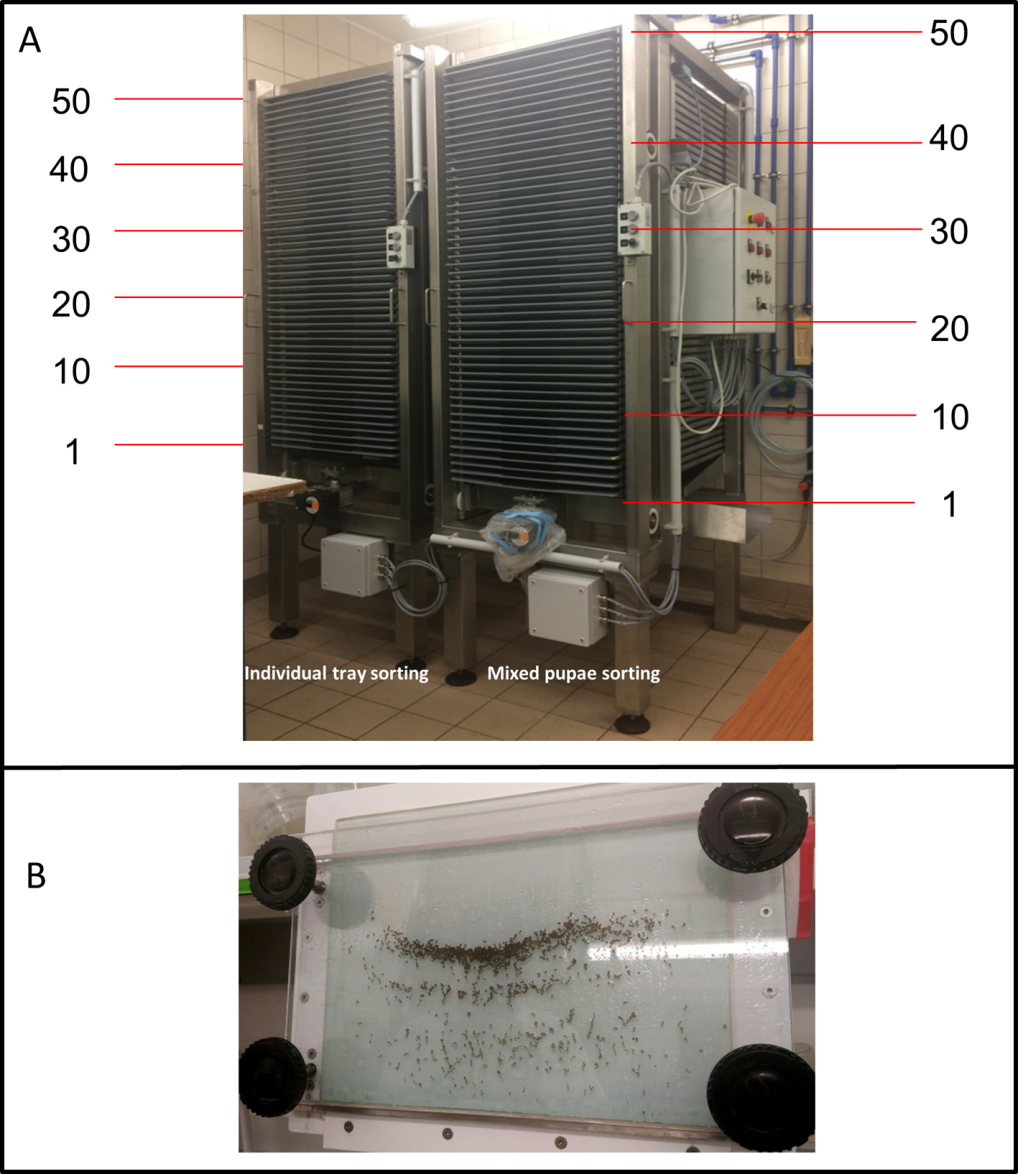
(A) The FAO/IAEA larval rearing unit with the experimental selected larval trays positions and (B) the Fay–Morlan glass plate separator.

*Aedes aegypti* mosquitoes used in this study originated from Juazeiro, Brazil (provided by Moscamed, IAEA Collaborative Center) and has been maintained since 2012 at the IPCL of the joint FAO/IAEA Division of Nuclear Techniques in Food and Agriculture, Seibersdorf, Austria, under controlled temperature (T), relative humidity (RH) and light regimes: the larval rearing room is maintained at 28 ± 2 °C, 80 ± 10% RH and the adult rearing room at 26 ± 2 °C, 60 ± 10% RH with 11:1:11:1 h light:dusk:dark:dawn photoperiod. This experiment was carried out in a large climate-controlled room where temperature and humidity were maintained at 30 ± 1 °C, 70 ± 10% RH. Eggs were collected from mass-rearing cages and dried following procedures developed at the IPCL [3, 16, 30]. Egg batches (2 weeks-old) were gently brushed from the egg papers. Three samples of 100–150 eggs were used to check the hatch rate. Based on the egg hatch rate, egg batches corresponding to 18,000 first instar larvae were quantified following the method described by Zheng et al. [51] and then hatched separately in jam jars (IKEA of Sweden AB SE-343 81 Almhult, Germany) containing 700 mL of boiled-cooled osmosis water and 10 mL of 4% IAEA diet (see [16, 30] for details). After hatching (from 2 PM to 9 AM, approximately 20 h, see Table 1), the contents of jars (first instar larvae) were sieved (50-μm sieve, Retsch^®^ Test Sieve with steel mesh) and transferred into mass-rearing trays (*L* × *W* × *H* = 100 × 60 × 3 cm, Glimberger Kunststoffe Ges.m.b.H., Austria) at selected trays within the rack as shown in Figure 1. Independent of the selected trays and to simulate real conditions, all of the fifty trays were filled with five liters of osmosis water one day before the addition of larvae. Larvae were fed with 4% (wt/vol) larval food suspension composed of 50% tuna meal, 35% bovine liver powder and 15% brewer’s yeast provided daily in the following amounts: 50 mL on day 1, 100 mL on day 2, 200 mL on day 3 and 4, 150 mL on day 5 and 50 mL from day 6 onwards [16]. The temperature of the water in the selected trays and of the room was recorded daily at 9 AM from first day of introducing L1 until pupation using a contactless clinical infrared thermometer (Geratherm^®^ CE0197, Germany). Pupae were harvested at 9 AM on five consecutive days, from day 6 to day 10 after hatching. The first collection was done at approximately 20 h from the beginning of pupation. Trays contents were collected either individually (for “individual tray sorting”) or all together by tilting the rack (for “whole rack sorting”). After tilting, the larvae-pupae mix was divided equally into six batches. Larvae and pupae for each tray were sieved by using a 600-μm sieve (Retsch^®^ Test Sieve with steel mesh) and transferred into small trays for sorting. Trays were assigned randomly to three different operators (the assigned trays were changed between operators during the following replicates, so that all operators sorted trays at all positions) and sorting of larvae, male and female pupae was performed mechanically using a Fay–Morlan glass separator [17] as redesigned by Focks (John W. Hock Co., Gainesville, FL, USA) [18]. After sorting, the remaining larvae were returned to the original rearing tray with the same used larval rearing water (with a daily volume reduction of 0.5 L). For each tray, male and female pupae were estimated volumetrically using a modified tube following standard operating procedures developed at the IPCL [16]. Male (or female) pupae recovery rate was calculated as the ratio of male (or female) pupae collected and the initial number of male (or female) larvae considering equal numbers of males and females in the initial number of larvae. From male pupae batch, three samples of 100 pupae of each tray were checked and sex verified by observing the genitalia of the pupae under a stereomicroscope [36] to assess the female pupae contamination rate.

**Table 1:** Rearing schedule with the main tasks and time used in this experiment.

Days	Main tasks and time
Day −1 (Monday)	Brush eggs Sample (100–150 eggs/sample) and hatch (2 PM) Prepare jars with boiled osmosis water
Day 0 (Tuesday)	Determine the egg hatch rate Weigh eggs Hatch eggs in jars (2 PM) Prepare trays with 5 L of osmosis water
Day 1 (Wednesday)	Transfer the content of hatching jars (L1) to mass-rearing trays (9 AM) Feed larvae (9 AM)
Day 2 (Thursday)	Feed larvae (9 AM)
Day 3 (Friday)	Feed larvae (9 AM)
Day 4 (Saturday)	Feed larvae (9 AM)
Day 5 (Sunday)	Feed larvae (9 AM)
Day 6 (Monday) – Day 9 (Thursday)	Tilt the trays (9 AM) Sort larvae and pupae (10–12 AM) Estimate the number of pupae by sex Check female contamination rate Pour the larvae back into the trays and feed (12 AM)
Day 10 (Friday)	Tilt the trays (9 AM) Sort larvae and pupae (10–12 AM) Estimate the number of pupae by sex Check female contamination rate

### Statistical analysis

Statistical analyses were performed using R Software [39]. A Gaussian linear mixed-effects model was used with number of pupae collected and temperature assigned as response variables, day of tilting and tray position as fixed effect, and replicate as a random effect [24] and when necessary, ANOVA was used for mean comparison. We also used binomial generalized linear mixed models fit by maximum likelihood (Laplace approximation) with the female contamination rate as response variable, tray position, and type of tilting, the operator and day of pupation as fixed effects, and the replicate as a random effect.

## Results

### Tray water temperature during rearing and across tray positions inside the rack

**Figure 2:**
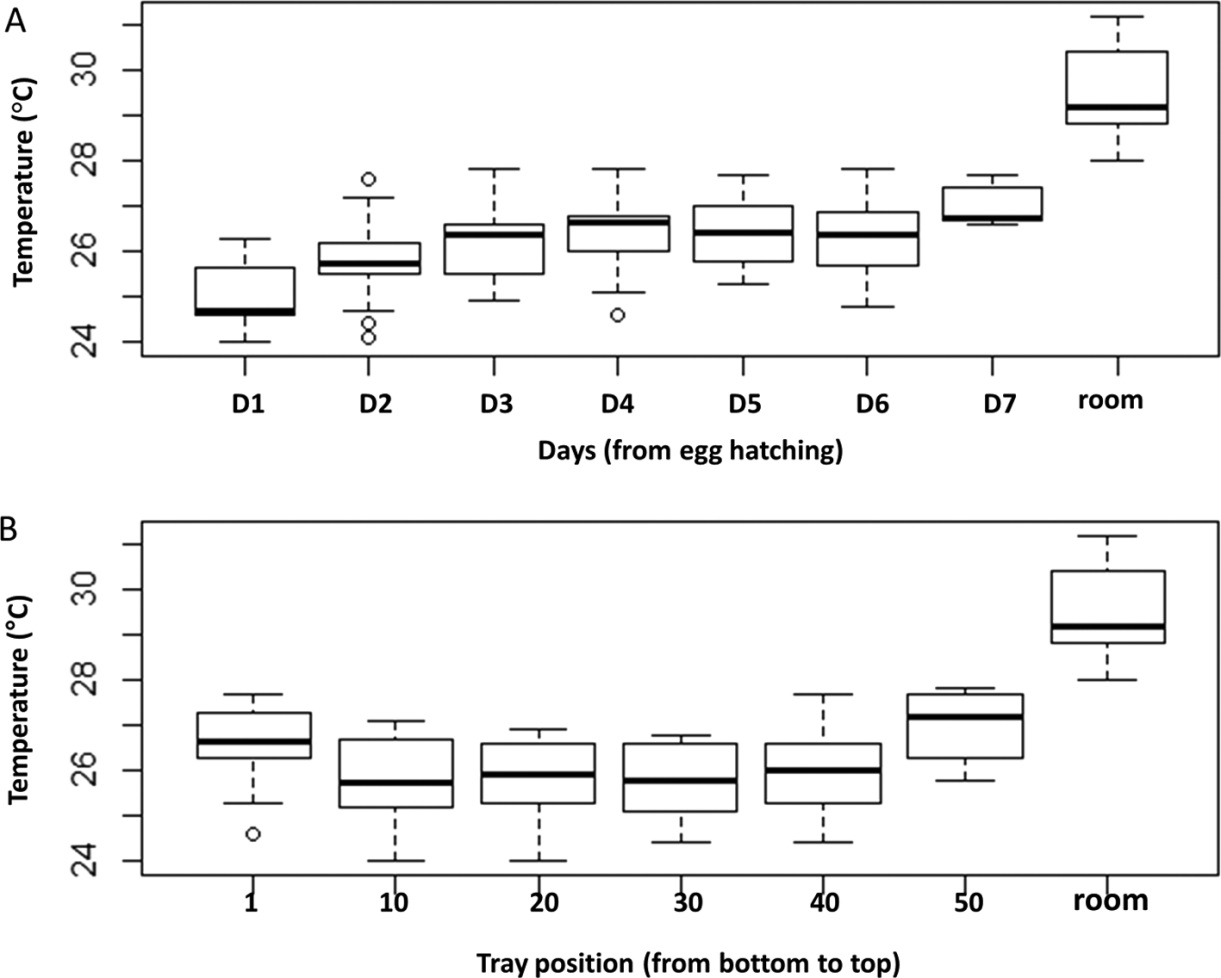
(A) Rearing water temperature variation during the course of rearing and (B) across the positions of the tray inside the rack. Water temperature increased significantly with time of rearing and between trays within the rack. Results are expressed as mean ± SE.

Data on temperature (rearing room temperature and water rearing temperature in the trays from bottom and top) during the rearing are shown in Figure 2. Water temperature within trays ranged between 24.0 °C and 27.8 °C, with a mean water temperature throughout the rearing duration of 26.2 °C. Overall, water temperature increased significantly with time of rearing (Fig. 2A, *df* = 7, *F* = 59.83, *p* < 2e-16).The initial temperature of water coming directly from the tap was around 22 °C and after 24 h, i.e. the first day when first instar larvae were introduced, the temperature was around 24 °C and then increased from day 2 to day 3 before stabilizing and then reaching the maximum on day 7 (27.8 °C). However, the temperature of water significantly varied between trays according to their position in the rack (*df* = 6, *F* = 74.74, *p* < 2e-16). Water in the bottom and in the top trays of the rack rearing unit had a higher temperature than the middle trays (Fig. 2B). The mean temperatures were 26.61 ± 0.16 °C, 25.83 ± 0.16 °C, and 27.02 ± 0.14 °C for the bottom tray, middle trays, and the top tray, respectively. The daily air temperature of the rearing room varied between 28.8 °C and 31.2 °C during the whole period, which was always higher than water temperature.

### Pupal production over five days of collection

**Figure 3:**
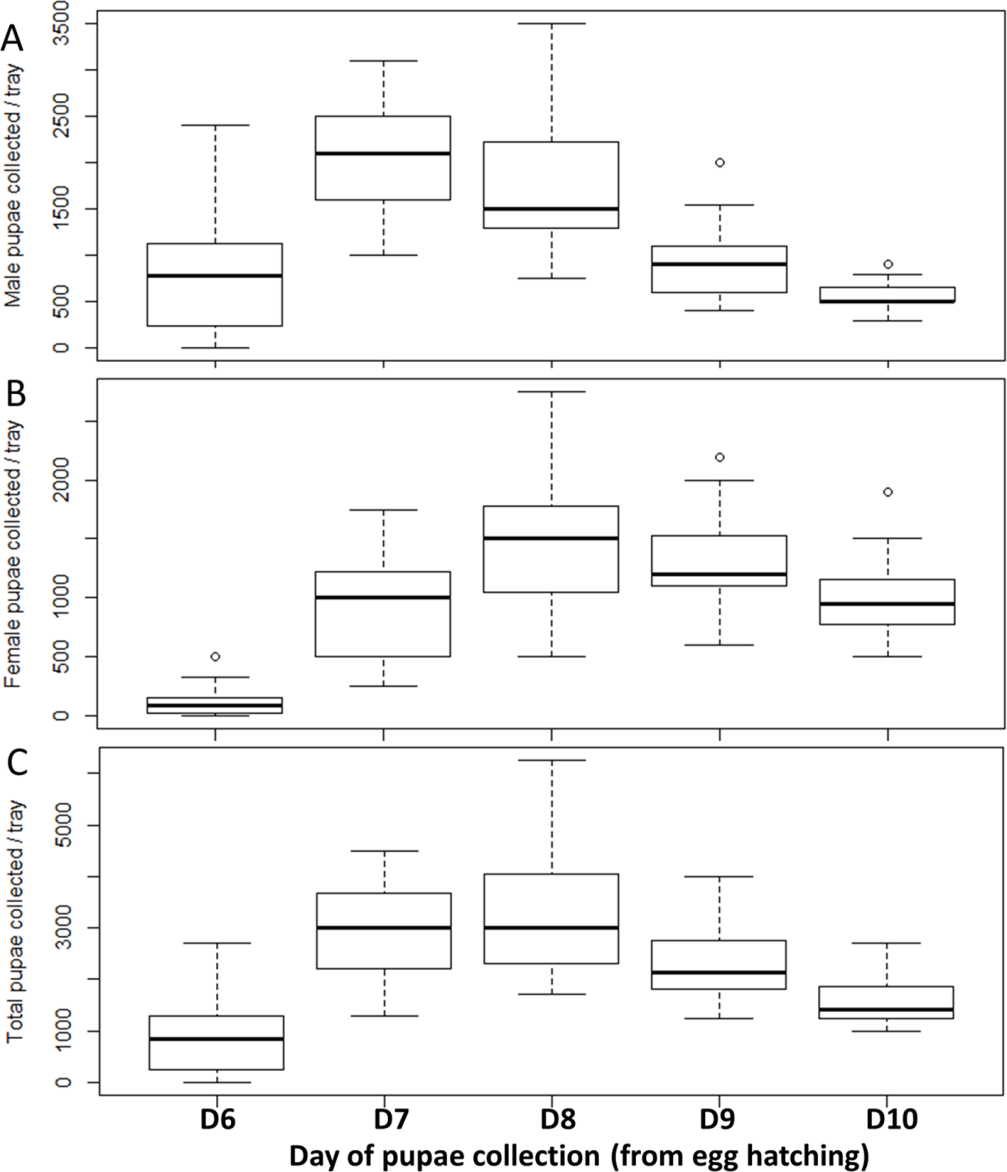
Number of (A) male pupae, (B) female pupae, and (C) total pupae as a function of the day of pupae collection in *Aedes aegypti.* Significant variation in the number of pupae collected as a function of the day of collection. Data presented in the figure are expressed as mean ± SE.

The numbers of male pupae, female pupae, and total pupae collected on a daily basis (at 9 AM every day) over five consecutive days are presented in Figure 3. There was significant variation as a function of the day of collection in the number of male pupae (*df* = 4, *F* = 55.09, *p* < 2e-16), female pupae (*df* = 4, *F* = 70.18, *p* < 2e-16) and total pupae collected (*df* = 4, *F* = 49.95, *p* < 2e-16). The number of pupae at the first collection was significantly lower (8.86% and 1.14% recovery for males and females, respectively). Overall, the number of male pupae at the second collection (D7) and female pupae at the third collection (D8) were greater (22.83% and 16.3% of recovery for males and females, respectively) in comparison to D6, D9, and D10 (Fig. 3A).

Based on the pupae obtained from a single tray, the production of the whole rack was estimated and the results are summarized in Table 2. Each rack produced about 305,219 ± 20,324 male pupae within five collections. However, limiting the pupae collection to 44 h from the onset of pupation, one rack produced about 142,720 ± 10,039 male pupae. The maximum male pupae collected occurred between the second and the third collection (approximately 62% of the male production capacity).

**Table 2:** Rack pupal production estimation in *Aedes aegypti* using the FAO/IAEA larval diet and feeding regime.

	Day 6	Day 7	Day 8	Day 9	Day 10
Male pupae/tray	798 ± 106	2055 ± 94	1725 ± 112	961 ± 67	564 ± 27
Male recovery (%)	08.20 ± 1.66	22.04 ± 1.26	17.35 ± 1.66	09.57 ± 0.88	06.51 ± 0.43
Female pupae/tray	103 ± 18	870 ± 68	1467 ± 89	1308 ± 68	1000 ± 54
Female recovery (%)	01.16 ± 0.35	09.23 ± 1.12	13.52 ± 1.11	13.18 ± 1.12	10.43 ± 0.70
Estimated male pupae/rack	39,943 ± 5321	10,277 7 ± 4718	86,250 ± 5603	48,055 ± 3348	28,194 ± 1334
Estimated female pupae/rack	5155 ± 899	43,542 ± 3424	73,333 ± 4452	65,417 ± 3410	50,000 ± 2716

### Pupal production from different positions of the tray in the rack

**Figure 4:**
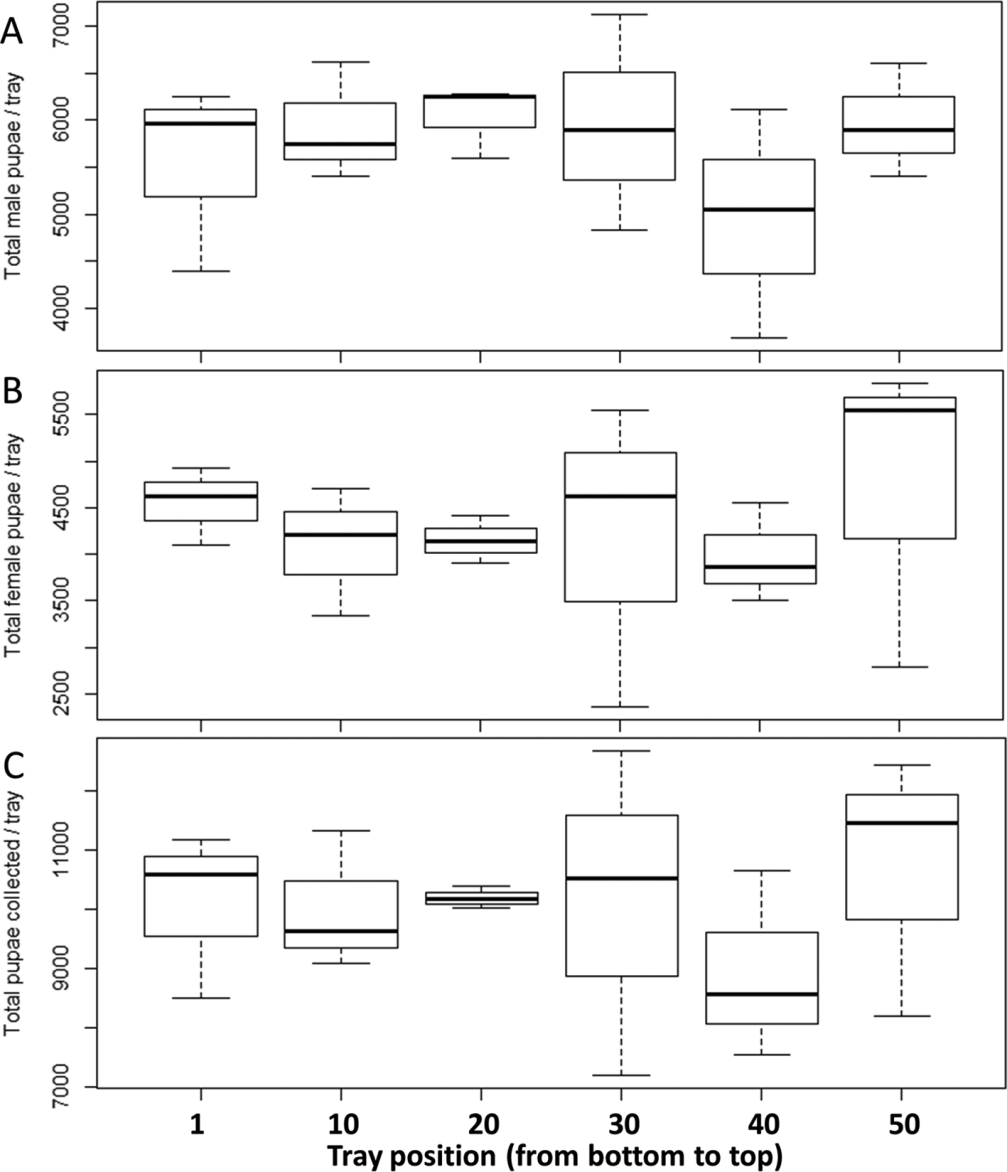
Number of (A) male pupae, (B) female pupae, and (C) total pupae as a function of the position of the rearing tray inside the rack in *Aedes aegypti.* No significant variation in the number of pupae collected as a function of tray position within the rack. Data presented in the figure are expressed as mean ± SE.

Regardless of tray position in the rack, pupation began on the 6th day after egg hatch, and pupae were harvested on the 6th, 7th, 8th, 9th, and 10th day. There was no significant difference in the number of male pupae (ANOVA, *df* = 5, *F* = 0.682, *p* = 0.646), female pupae (ANOVA, *df* = 5, *F* = 0.237, *p* = 0.939) and total pupae (ANOVA, *df* = 5, *F* = 0.332, *p* = 0.884) collected from the different positions of trays in the rack although more pupae were generally collected from the bottom and top trays the first day of collection (Fig. 4).

### Factors influencing the efficiency of sex sorting: female contamination rate in male pupae

**Figure 5:**
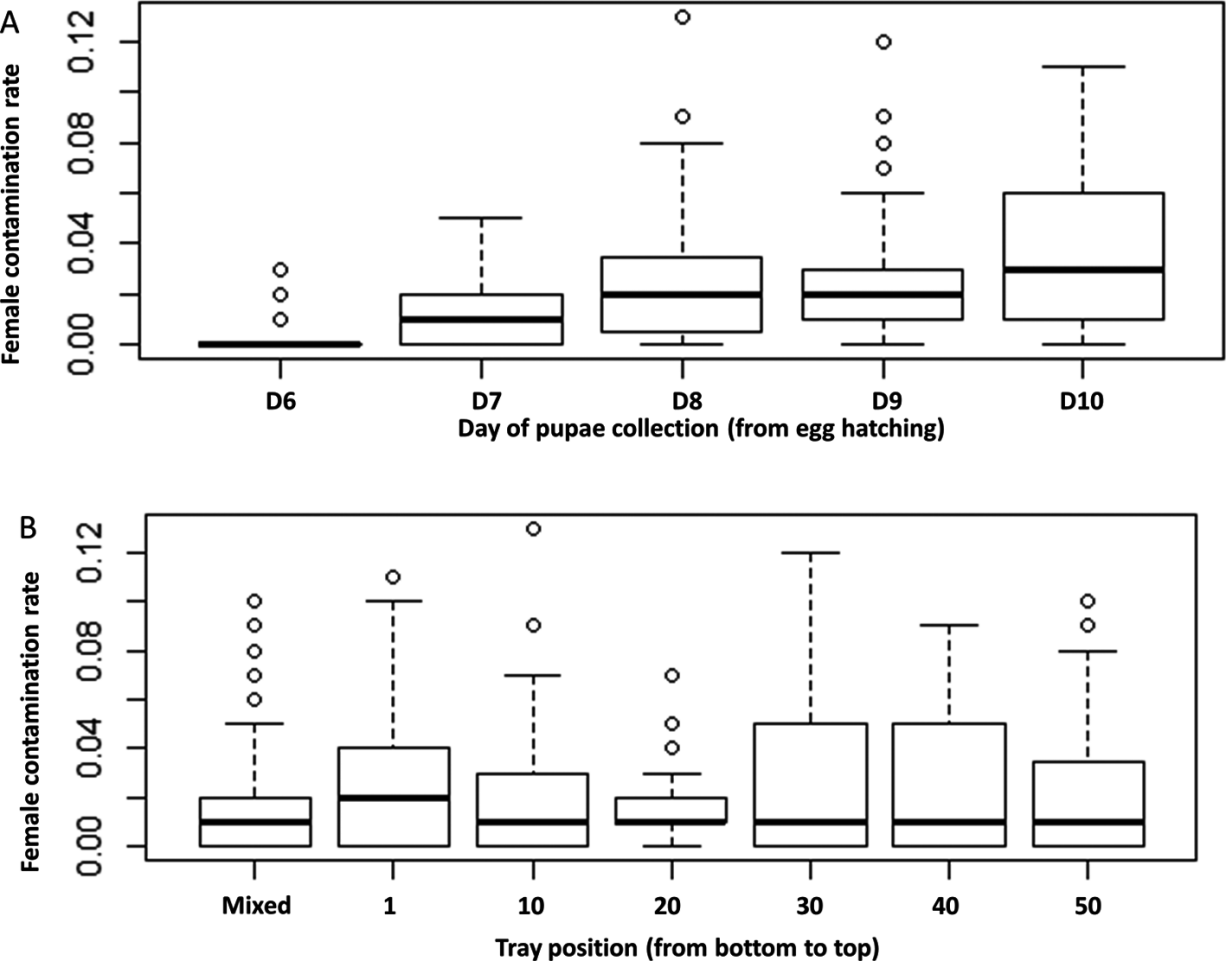
Female contamination rate as (A) a function of the day of pupae collection and (B) the position of the rearing tray inside the rack in *Aedes aegypti.* Significantly increased female contamination rate over the course of sorting days. Data presented in the figure are expressed as mean ± SE.

**Figure 6:**
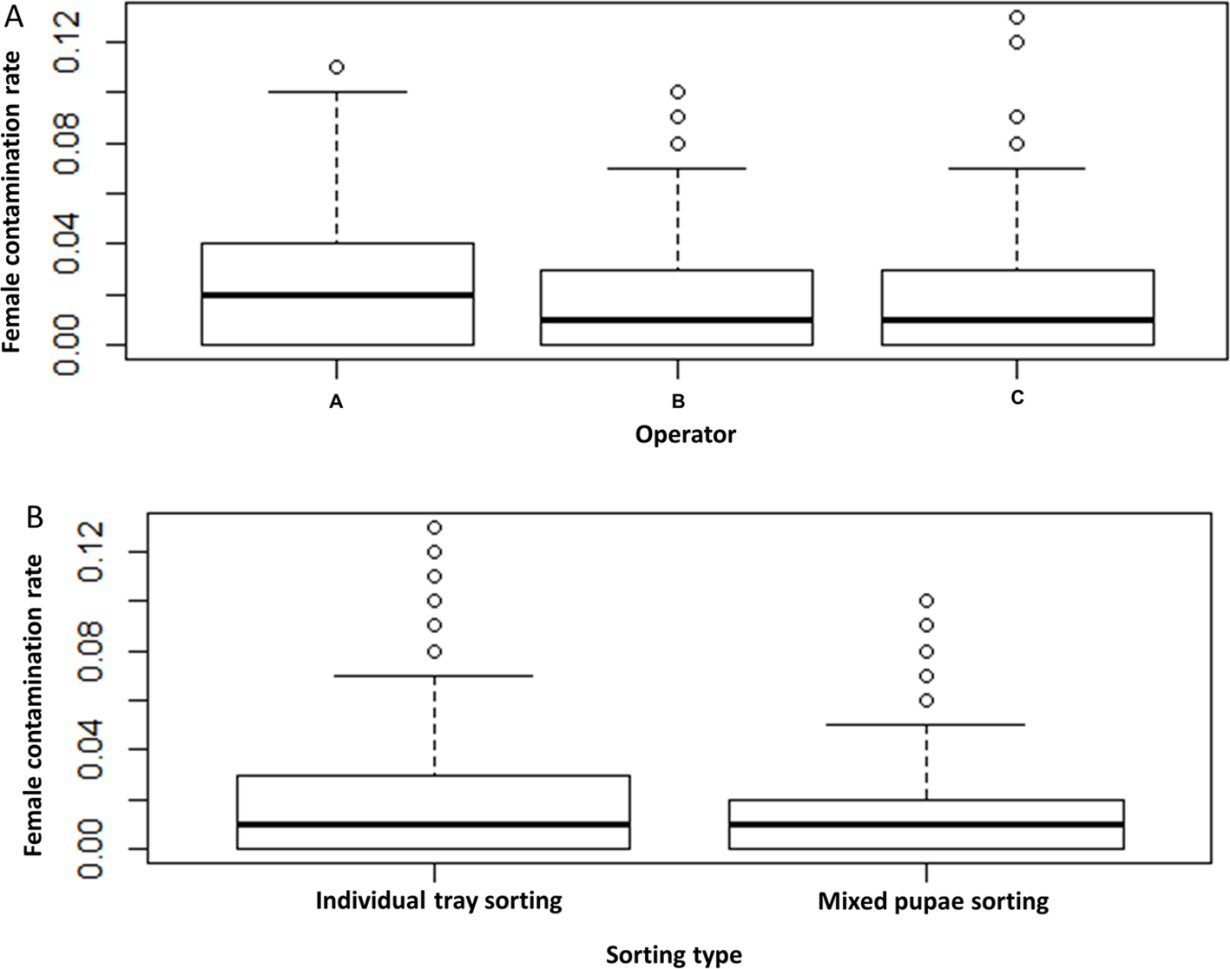
Female contamination rate as (A) a function of the operator and (B) sorting type in *Aedes aegypti.* Significant variation in female contamination rate between operators. Data presented in the figure are expressed as mean ± SE.

The percentage of female pupae in male pupae batches collected after sorting was determined with regard to the position of the rearing tray inside the rack, the day pupae were collected, the individual who operated the sorting and the method of sorting pupae, i.e. “tray by tray or all tray contents mixed”. Results are shown in Figures 5 and 6 and the statistical analyses in Table 3. In all cases, the female contamination rate ranged from 0.0023 ± 0.0006 to 0.0371 ± 0.0028 (mean ± SE). The female contamination rate significantly increased over the course of sorting days. When compared to the first collection (D6), female contamination rates were significantly higher in collections 3 (D8), 4 (D9), and 5 (D10), but no difference was found between first (D6) and second (D7) collections (*z* = 0.848, *p* = 0.396). The female contamination rate significantly varied with the operator (Table 3, *p* < 0.05). As a consequence of pupae loss, the male, female, and total pupae recovery rate differed between operators as shown in Table 4. Neither tray position within the rack nor type of sorting significantly influenced the female contamination rate (Table 3, *p* > 0.05).

**Table 3:** Results of the binomial generalized linear mixed model for the effect of day of sorting, tray position in the rack, type of sorting, and the operator on female contamination rates in male pupae using the Fay–Morlan glass plate separator.

Factors	Estimate	SE	*z*-value	*p*-value
(Intercept)	−5.071	0.44928	−11.287	<2e-16
Position within the rack				
Tray position 10	−0.05583	0.73281	−0.076	0.939
Tray position 20	0.05491	0.67326	0.082	0.9349
Tray position 30	−17.23784	2328.5055	−0.007	0.9941
Tray position 40	−0.64143	0.83862	−0.765	0.4443
Tray position 50	−17.25053	2853.79142	−0.006	0.9952
Type of sorting				
Mixed pupae sorting	−1.03663	0.83814	−1.237	0.2162
Day of sorting				
Day 7	0.47505	0.55998	0.848	0.3962
Day 8	1.3836	0.4979	2.779	**0.0054**
Day 9	1.70302	0.48558	3.507	**0.0004**
Day 10	1.80168	0.48246	3.734	**0.0002**
Operator				
Operator B	−0.24502	0.07293	−3.36	**0.0008**
Operator C	−0.28459	0.07593	−748	**0.0001**

Values were compared to the reference tray position 1 (bottom), individual tray sorting, day 6 and operator A. Values in bold indicate a statistically significant difference (*p* < 0.05).

**Table 4:** Estimated male, female, and total pupae recovery rates and loss of larvae between operators.

Operator	Male pupae recovery (%)	Female pupae recovery (%)	Total pupae recovery (%)	Estimated loss of larvae (%)
A	58.29 ± 05.18a	47.33 ± 02.64a	52.81 ± 03.69a	47.19 ± 3.69a
B	66.70 ± 02.25b	49.30 ± 05.57a	58.00 ± 03.57a	41.99 ± 3.57a
C	65.98 ± 04.08b	45.59 ± 05.60a	55.70 ± 04.70a	44.05 ± 4.70a

Within a column, different letters indicate a statistically significant difference (*p* < 0.05).

## Discussion

The SIT relies on mass-rearing the target species, and in the case of mosquitoes, requires male-only releases. Its operational success partly depends on the capacity to produce males in sufficient numbers to achieve appropriate sterile-to-wild male ratios [15, 40] and an efficient system allowing the elimination of females before male releases. The Joint FAO/IAEA has developed mass-rearing equipment such as the tray-rack unit for rearing immature mosquito stages and this has been tested for *Ae. albopictus* [4, 49] and *An. arabiensis* [33]. In this study, we aimed at exploring the use of this rearing unit for producing*. Ae. aegypti* male pupae and attempted to identify potential factors that could impact its productivity and the efficiency of sex sorting using the Fay–Morlan glass separator.

With the rearing procedures described in the present study, the FAO/IAEA larval rearing unit can be used successfully for mass-rearing *Ae. aegypti* mosquitoes for large-scale SIT application. Although pupae production was lower when compared to that of *Ae. albopictus* as shown by Balestrino et al. [4] and Zhang et al. [49] using the same rearing unit and larval density but with different diets and feeding regimes, it was possible to produce up to 300,000 male pupae per rack over five consecutive days of tilting/sorting. This pattern of daily pupae production, which significantly varied between the collection days, indicates large developmental plasticity. As a consequence of this plasticity, pupation occurred over several days. This extended pupation time window may be attributed either to the lack of hatching synchronization, the higher larval density, or to insufficient larval food reducing faster and synchronized development and thus pupation. Puggioli et al. [38] have demonstrated that diet concentration significantly affected the survival time to pupation. Studies on the optimization of larval feeding using new ingredients such as black soldier fly larvae powder in replacement of costly bovine liver powder [8, 32] will need to be explored further.

In mass-rearing settings, it is important to minimize space between trays while balancing against the need to deliver air and regulate temperature. This study showed no significant difference in pupae production when trays were stacked at the top, middle, or bottom, reflecting homogenous rearing between the different trays within the rack. Similar female contamination rates observed across tray positions further support this finding. These results are consistent with those observed in China with *Ae. albopictus* using the same rack [49]. On the other hand, water temperature, a key variable that affects mosquito development should be considered when rearing mosquitoes. Significant variation between tray positions and the increase over the course of the study must be taken into account. Insects can only thrive within a range of temperatures and thus, beyond critical minimum and maximum points, their survival and activity is affected, as well as the speed of their development [14]. Higher temperature was found in the upper and the bottom trays. However, this did not affect the rearing outcomes, probably because the variation still remains in the optimum rearing temperature range of *Aedes* mosquitoes of 26–28 °C [4].

For SIT-based approaches or any other population suppression approach, the elimination of female mosquitoes prior to male releases is not only essential, but mandatory for its application. Due to the lack of a perfect sex sorting method, many sex sorting methods are being used such as metal sieving plates [5, 19, 34, 41], computer vision analysis [48], and the Fay–Morlan glass separator. More consideration should therefore be given to the efficiency of these methods and to factors that could affect the result, so that measures can be taken to avoid the release of females in the field. To the best of our knowledge, available data on the use of the Fay–Morlan glass separator for *Ae. aegypti* pupae sex separation were only based on small-scale rearing (750–2000 larvae per rearing tray). Recorded female contamination rates differed from one setting to another: 0.1% in Thailand [22] to 16% in Sri Lanka [19], both in *Ae. aegypti*. Different rearing protocols (larval density, feeding regime) and the selection of males and females for colony could induce different selection pressures and thereby lead to pupal size variations or different degrees of protandry. In addition, the volume of larvae/pupae mixture introduced into the system by the operator, the diet composition, or even the ability to determine the sex can be suggested as potential factors to explain the difference in female contamination between laboratory settings. Regardless of the responsible factor, this highlights the need to develop automated and efficient sorting methods to standardize the separation process, ensuring reasonably consistent sex separation with an acceptable level of female contamination for large-scale release operations. Our results showed that the efficiency of sex sorting was negatively impacted when pupae were collected several times (days) after the first collection. The female contamination on male pupae was very low (0.23%) for the first pupae collection, which is a good result for any program release. Release of a population with more than one percent of females which cause nuisance and transmit pathogens could worsen an epidemic and is not acceptable; only <1% female contamination can be tolerated [21, 27]. This 1% female contamination rate concerns the adult stage, i.e. the released sterile males. The female contamination rate should be reduced to a minimum and this threshold is only indicative. It should actually not exceed a predetermined threshold agreed with the public health authorities [21]. It was not surprising to find this result due to the protandry phenomenon that exists naturally in *Aedes* mosquitoes. Hence, protandry, defined as the earlier sexual maturation of males compared to con-specific females [6, 27], contributed to the low female contamination rate observed in pupae collected within 20 h from onset pupation, due to the small number of female pupae present. The sex ratio of these pupae collected at 20 h from the beginning of pupation (day 1) was 87.89% males and 12.11% females. However, we found a female contamination rate higher than 2% from the collections on days 3 to day 5. Among pupae collected on the subsequent days, the total percentage of female pupae increased from 29.38% (day 2) to 61.27% (day 5). These males and females that matured simultaneously may have developed to an intermediate size with reduced pupal size dimorphism [6] and therefore overlapping in size and leading to an increased female contamination rate. Larval food and the rearing medium could not be good enough to induce a suitable sexual dimorphism or some external stimuli such as multiple titling events might have increased the pupation speed of larvae as they face danger, and the early formed female pupae might have a smaller size. Therefore, one rack tilting event or maximum two is acceptable for male production for a minimum female contamination rate in this case. On the other hand, the operator of the larval–pupal glass separator clearly influenced the efficiency of sex sorting. This is not surprising, because manual handling of the sex sorter is labor intensive, time consuming, and can cause fatigue. It requires skills to adjust the slope of the outer glass and a high level of attention by the operator to control the collection of each batch (larvae, male, and female pupae) without collecting the male and female pupae that overlap in size. Therefore, developing an automated system has become a necessity and would offer faster, more accurate and reproducible results. However, neither the position of the tray within the rack nor the type of collection pupae influenced the pupation rate and female contamination rate. This is particularly important in the context of mass-rearing and suggests that all the trays allow homogeneous larval development, and produce uniformly sized pupae in the different trays within the rack, thereby supporting consistent sex sorting. Based on the recommendation that only sex sorting methods with a female contamination rate of less than 1% is acceptable by the public and legislators for field releases [1, 21, 27], our results revealed that only pupae collected within 20 h from pupation onset (day 1) or 44h (day 2) sorted by well-trained operators can achieve this goal. We recommend that only pupae recovered on days 1 or 2 of pupation (corresponding to a 30% male recovery rate with this protocol) should be used for releases, and that persons operating the sexing system should be well trained. Quality assurance measures should be in place to verify female contamination rates and re-sorting is an option for instances where more than 1% females are detected.

## Conclusion

Data obtained in this study demonstrated the suitability of using the FAO/IAEA reference larval rearing unit for mass-rearing *Ae. aegypti* mosquitoes, without any impact of the tray position within the rack on pupal production and sex sorting efficiency. However, it highlights the need to optimize the feeding regime to ensure a high male recovery rate for the first days of pupation coupled with low female contamination rates and to take into account factors such as the operator that can affect the sex sorting efficiency when using the Fay–Morlan glass separator. Evaluating the production capacity of the larval mass-rearing technology can contribute to decision-making processes associated with facility design, construction, costing, and operation for the overall SIT operational plan.

## Data Availability

All data generated or analyzed during this study are included in this published article.
